# Elucidating the Probiotic Potential of *Lactobacillus* and *Bifidobacterium* Species in Modulating Glucose Metabolism and Inflammation in Gestational Diabetes Mellitus: A Scoping Review

**DOI:** 10.1093/nutrit/nuaf238

**Published:** 2025-12-10

**Authors:** Sidra A Uzair, Angela Ajeesh, Salma H Ahmed, Shaikha Alabduljabbar, Fatima Ahmad, Arun P Lakshmanan, Annalisa Terranegra

**Affiliations:** Translational Medicine Department, Sidra Medicine, Doha 26999, Qatar; Translational Medicine Department, Sidra Medicine, Doha 26999, Qatar; Translational Medicine Department, Sidra Medicine, Doha 26999, Qatar; Translational Medicine Department, Sidra Medicine, Doha 26999, Qatar; Translational Medicine Department, Sidra Medicine, Doha 26999, Qatar; College of Health and Life Sciences, Hamad bin Khalifa University, P.O. Box 34110, Doha, Qatar; Translational Medicine Department, Sidra Medicine, Doha 26999, Qatar; Translational Medicine Department, Sidra Medicine, Doha 26999, Qatar; College of Health and Life Sciences, Hamad bin Khalifa University, P.O. Box 34110, Doha, Qatar

**Keywords:** probiotics, *Lactobacillus*, *Bifidobacterium*, GDM, glucose metabolism, inflammation

## Abstract

Gestational diabetes mellitus (GDM) affects 14% of pregnancies globally, contributing to gut microbial dysbiosis, inflammation, and insulin resistance. Probiotic supplementation with *Lactobacillus* and *Bifidobacterium* species has potential in addressing GDM by modulating gut microbiota. However, findings from clinical trials are inconsistent. In this scoping review, the evidence on the role of *Lactobacillus-* and *Bifidobacterium*-based probiotic supplementation in managing GDM is mapped systematically, and knowledge gaps and future research directions are identified. Following Arksey and O’Malley’s framework, an initial database search was conducted in February 2023 across 4 literature databases for articles on randomized controlled trials published since 2013; a second search in January 2025 included studies published after February 2023, reflecting ongoing research in this field. Title and abstract screening were conducted using Rayyan, followed by full-text review, yielding 29 eligible studies. Cohen’s *d* was used to quantify the magnitude of the effect. The interventions included encapsulated probiotics or fermented foods containing various *Lactobacillus* and *Bifidobacterium* species. The findings consistently showed improvements in fasting glucose levels, insulin sensitivity, and reduction in serum insulin levels and homeostatic model assessment for insulin resistance with increased Quantitative Insulin Sensitivity Check Index scores. Improvements in glucose metabolism were linked to short-chain fatty acids (SCFAs), activating G-protein coupled receptors (GPRs), boosting GLP1 secretion, and insulin release. Probiotic interventions also favorably modulated inflammatory markers (high-sensitivity C-reactive protein, interleukin-6 [IL-6], and tumor necrosis factor-α), likely through enhanced mucus production, SCFAs binding to GPR109, strengthening tight junctions, and concurrent release of IL-18 and IL-10. The evidence indicates the potential of *Lactobacillus-* and *Bifidobacterium*-based probiotic interventions to beneficially influence glucose metabolism, inflammatory response, and gut microbial balance in GDM. Heterogeneity in probiotic formulations across studies, probiotic doses, and regional differences in diet and lifestyle underscore the need for more targeted and standardized research to fully understand the therapeutic potential of probiotics in GDM.

## Graphical Abstract

**Figure:**
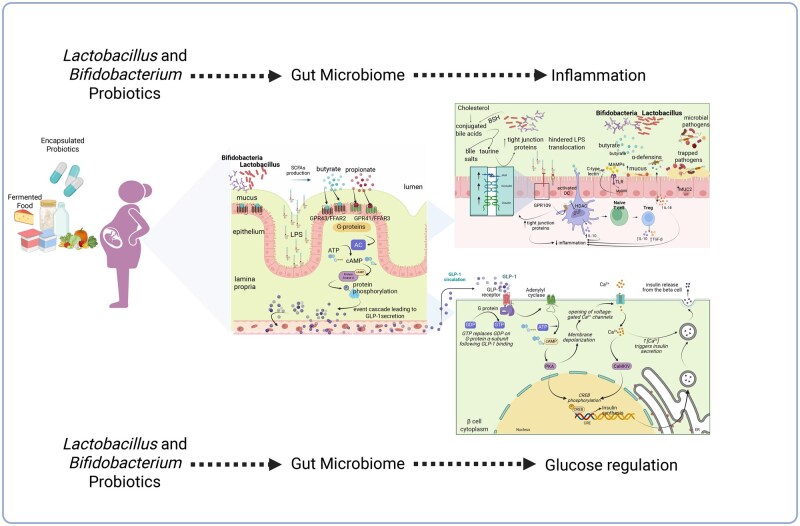

## INTRODUCTION

Gestational diabetes mellitus (GDM) is a public health concern affecting 14% of pregnancies globally^1^ and is associated with several short- and long-term adverse outcomes, such as an increased risk of preeclampsia,^2^ a 10-fold increased risk of developing type 2 diabetes mellitus (T2DM) and cardiovascular disease,^3^ with a 2- to 4-times increased risk of developing antenatal or postpartum depression.^4^ For offspring, GDM can result in complications in childbirth, such as infants being large for gestational age^2^ and preterm births.^5^

Unhealthy dietary choices and an inactive lifestyle are the 2 most important potentially modifiable factors for GDM.^6^ A healthy, balanced diet reduces the risk of GDM by 15%-38% when it is characterized by minimally processed, plant-based foods incorporating all essential food groups, and scoring higher in terms of quality, or is based on guidelines of Dietary Approach to Stop Hypertension.^6^ Similarly, the Mediterranean diet—characterized by a higher consumption of plant foods, including fruits, vegetables, legumes, nuts, seeds, whole-grain cereals; a moderate consumption of dairy products; and a low-to-moderate consumption of foods containing animal protein, such as red meat, fish, poultry, and eggs^7^—reduced the risk of developing GDM^8–10^ and inflammatory biomarkers such as high-sensitivity C-reactive protein (hs-CRP)^11^ while increasing the levels of fiber-degrading *Faecalibacterium prausnitzii,*^11^ and subsequent metabolites, such as 2-methyl butyrate.^12^ On the contrary, greater consumption of foods such as unprocessed or processed meat and potatoes, which contribute to increased saturated fats and calorie intake,^6^ as well as body mass index associated with overweight and obesity, increases the risk of GDM^13^^,^^14^ while simultaneously resulting in epigenetic alterations during pregnancy, affecting cord blood and placenta.^15^ A high-fat diet has been associated with microbial dysbiosis, causing an abundance of gram-negative *Proteobacteria,*^16^ which produce endotoxin and lead to increased levels of postprandial lipopolysaccharide (LPS) and pro-inflammatory biomarkers.^17^ Lack of dietary fiber in the diet decreases fermentable short-chain fatty acids (SCFAs), reducing endogenous glucagon-like peptide-1 (GLP-1) and insulin secretion.^18^ This is compounded by a lack of physical activity during pregnancy, because 23%-29% of women remain inactive and do not meet the physical activity recommendations for pregnancy.^19^ Thus, the primary therapeutic intervention in addressing GDM revolves around addressing this duo. A dietary approach to treating GDM involves curtailing refined carbohydrates, fats, and added sugars while including whole grains and dietary fiber^20^ to improve the production of beneficial microbial metabolites, such as SCFAs. Additionally, a moderately active lifestyle during pregnancy contributes positively toward maintaining the recommended sugar levels during gestation.^20^

The human gut is home to millions of microbes that confer health benefits to the host by protecting the intestinal epithelium and strengthening the immune response.^21^ A healthy, stable gut microbial community can play a significant role in colonization resistance against pathobionts. The composition of these microbes is heavily influenced by metabolic and hormonal changes during pregnancy,^22^ with a significant reduction in the α diversity and an increase in the β diversity.^23^ In the event of GDM, the microbiome composition undergoes substantial changes in the third trimester to resemble that of a nonpregnant woman with T2DM, with changes persisting until 8 months postpartum.^24^ Therefore, modifying gut microbial communities either through the intake of fermented food or probiotic supplementation can be a potential intervention to reduce the risk of GDM in pregnant women.^25^ According to the International Scientific Association for Probiotics and Prebiotics, probiotics are defined as “live microorganisms that, when administered in adequate amounts, confer a health benefit on the host.”^26^ This definition emphasizes 3 essential characteristics of probiotics: they must be microbial in origin, viable at the time of administration, and beneficial to health. Although various interventions have been explored for the management of GDM, a comprehensive meta-analysis of 46 randomized controlled trials (RCTs) involving 16 545 pregnant women identified probiotic supplementation as the most effective strategy to reduce the risk of developing GDM. It outperformed other interventions such as dietary modifications, physical activity, and inositol supplementation.^27^

A growing body of scientific evidence has emphasized the increased intake of food with high probiotic content or probiotics supplements to alleviate dysbiosis in the treatment of GDM, due to their safety in terms of not resulting in any adverse maternal and neonatal health outcomes,^28^ unlike glucose-lowering medicines, which might result in maternal and neonatal adverse outcomes.^29^ Selectively improving the gut microbiome may help control glucose homeostasis and improve insulin sensitivity and serum insulin levels during pregnancy.^30^ Reducing the harmful metabolic consequences linked to pathogenic bacteria colonization is the primary objective of using probiotics as part of a GDM therapy regimen.^31^ A dose of more than 10^7^ colony-forming units (CFU) probiotic counts per gram of the probiotic product with serving size of 100 g can have beneficial effects on metabolic profiles in pregnant women.^32^ Probiotic efficacy is strain specific, and a minimal effective dose should be confirmed in clinical studies. An effective dose typically ranges from 10^9^ to 10^10^ CFU per serving, consumed as fermented products or a supplement. Some multistrain probiotic supplements could have higher counts. The most widely used probiotics in improving glycemic control and insulin resistance include *Lactobacillus acidophilus*, *Bifidobacterium bifidum*, and *Lacticaseibacillus casei* (formerly *Lactobacillus casei*), which have been reported to favorably influence various glucose metabolic parameters, including fasting plasma glucose (FPG), insulin, homeostatic model assessment for insulin resistance (HOMA-IR), and quantitative insulin sensitivity check index (QUICKI).^33^

C-reactive protein is the most widely investigated biomarker of chronic subclinical inflammation in GDM; it causes insulin resistance by inhibiting the insulin signaling cascade.^34^ A typical diet devoid of food including fruits, vegetables, nuts, legumes, and whole grains results in reduced levels of gut commensals like *Faecalibacterium prausnitzii*, thereby causing the production of essential microbial anti-inflammatory molecules^35^ and bioactive molecules to plummet, which results in increased expression of inflammatory factors such as tumor necrosis factor-α (TNF-α) and diminished expression of interferon-γ (IFN-γ).^36^ Probiotic supplementation reduces gut permeability by hydrolyzing bile salt, which releases taurine, in turn leading to increased expression of tight junction proteins like occludin, claudin-1, and zonula occludens-1 (ZO-1), forming the tight junction complex, which regulates paracellular pathway.^37^ The gut microbiota–derived SCFAs inhibit histone deacetylase activity and activate G protein-coupled receptor pathways to reduce inflammation.^38^ However, not all probiotics reduce gut inflammation, which is highly strain dependent; hence, selectively targeted probiotics should be used to reduce gut inflammation.

Among all probiotics, *Lactobacillus* and *Bifidobacterium* species have been extensively studied for their safety and efficacy.^39^ They also form part of normal gut flora, have a long history of safe use, rarely cause infections in humans, and are beneficial. Compared with *Lactobacillus* and *Bifidobacterium,* the safety profile of other probiotics is not backed up by scientific research in the context of GDM. Considering the promising potential of *Lactobacillus* and *Bifidobacterium* species as safe therapeutic alternatives to mitigate metabolic disorders, there has been a significant increase in the consumption of probiotics,^40^ sourced either from food or in the form of supplements. However, some results have been controversial because some clinical trials using probiotics have reported no significant effect on the control of blood glucose and incidence of GDM. We chose to study probiotic use in GDM because the risk of acquiring noncommunicable diseases increases with women with GDM later in life,^3^ and compliance with recommended lifestyle factors in this population is low.^41^ In this scoping review, we compiled and critically reviewed the evidence of modulation of gut microbiota using *Lactobacillus* and *Bifidobacterium* species sourced through commercial preparations or fermented foods, thereby affecting glucose metabolism and inflammatory biomarkers in pregnant women with GDM.

## METHODS

The PICOS framework (Table 1) was used to formulate the research question for this scoping review. This framework also helped us define the inclusion and exclusion criteria, key words, and Medical Subject Heading descriptors and devise the search strategy for each database. The Preferred Reporting Items for Systematic Reviews and Meta-Analyses extension for Scoping Reviews (PRISMA-ScR) framework^42^^,^^43^ was followed to conduct a systematic search and to guide the review process. This systematic search aimed to critically analyze the evidence on the role of *Lactobacillus* and *Bifidobacterium* species in controlling blood glucose and inflammation in the defined population.

**Table 1.** PICOS Criteria for the Inclusion and Exclusion of Studies

Parameter	Inclusion criteria	Exclusion criteria
Population	Women with GDM or who develop GDM, regardless of ethnicity; studies published in 2013-2025	Women without GDM, or those who do not develop GDM, or nonpregnant women
Intervention	Single and multistrain probiotics in commercial preparations or fermented foods containing *Lactobacillus* and *Bifidobacterium* species	Any intervention other than probiotics or fermented foods
Comparator	Placebo or regular nonfermented food	
Outcomes	Improved glycemic outcomes and inflammatory biomarkers: FPG, 1-h OGTT, 2-h OGTT, serum insulin, HOMA-IR, HOMA2-IR, HOMA-β, QUICKI, serum hs-CRP, C-peptide, HbA_1c_, postprandial glucose, CatD, IL-1, IL-6, IL-8, IL-10, TNF-α, IFN-γ, MMP-8, IGFBP-1, phIGFBP-1, TGF-β, VEGF	All outcomes other than glucose metabolism and inflammation
Study design	Randomized controlled trials	Any other study types (ie, narrative reviews, scoping reviews, qualitative syntheses, systematic reviews, and meta-analyses of insufficient quality, cross-sectional studies, protocols of randomized controlled trials)

Abbreviation: CatD, cathepsin D; FPG, fasting plasma glucose; GDM, gestational diabetes mellitus; HOMA, homeostatic model assessment; HOMA2, updated homeostatic model assessment; hs-CRP, high-sensitivity C-reactive protein; IFN, interferon; IGFBP-1, insulin-like growth factor binding protein-1; IL, interleukin; IR, insulin resistance; MMP-8, matrix metalloproteinase-8; NS, not significant; OGTT, oral glucose tolerance test; phIGFBP-1, phosphorylated insulin-like growth factor binding protein-1; QUICKI, quantitative insulin sensitivity check index; TNF, tumor necrosis factor; VEGF, vascular endothelial growth factor.

### Inclusion and Exclusion Criteria

The target population included women experiencing GDM, regardless of age, ethnicity, weight, parity, pregnancy or prepregnancy body mass index, geographic location, and other comorbid conditions. The included studies recruited women who developed GDM during pregnancy and had no prior history of diabetes. Eligible interventions included food and commercially available supplements containing *Lactobacillus* and *Bifidobacterium* species. The review assessed the evidence from RCTs published since 2013, in English, with full text, that measured the impact of probiotic supplementation on biomarkers of glucose metabolism, including FPG, 1- and 2-hour oral glucose tolerance tests (OGTTs), serum insulin, HOMA-IR, HbA_1c_, and QUICKI. Included studies also measured the incidence of GDM. Studies analyzing the effect of inflammatory biomarkers such as hs-CRP, interleukins, TNF-α, and IFN-γ were also included (Table 1).

We included only RCTs because they are the gold standard to ascertain the efficacy and safety of any intervention.^44^ The rationale for choosing articles on RCTs published in the past 10 years was the emergence of and the momentum the field of gut sciences gained in the past decade. We included interventional studies testing either food containing probiotics or commercially available probiotics, which are recognized as safe,^45^ and their presence maintains gut health during pregnancy.^46^^,^^47^ The choice of English language was made to align with the reviewers’ familiarity with the language, thereby facilitating comprehension of the results from the included studies and the synthesis of the evidence.

Studies on animal models or with study designs other than RCTs (eg, prospective and retrospective comparative cohort studies, cross-sectional studies, reviews, case reports, records from grey literature, book chapters, conference proceedings) were excluded. Randomized controlled trials published before 2013 and in languages other than English were also excluded from the eligibility pool. Studies with a population of nonpregnant women or pregnant women not experiencing GDM could not meet the eligibility criteria. We used a snowballing process and carefully looked through the reference lists of the publications to find further pertinent research studies meeting the inclusion criteria.

### Data Sources, Search Strategy, and Study Selection

The search was carried out in February 2023 using 4 electronic databases—PubMed, Scopus, Web of Science, and Cochrane Library—for RCTs assessing the effect of probiotic interventions in improving biomarkers of glucose metabolism and inflammation. Medical Subject Heading terms were identified from the US National Library of Medicine, and relevant key words were listed (Table 2). The search strategy was then created by combining the Medical Subject Heading terms and key words using the Boolean operators “AND” and “OR.” Modifications were made in search strings to fit each database. The same filters of language, year of publication, study design, and accessibility were applied across all databases. A detailed search strategy for each database is outlined in Table 3. Given the possibility of continuous advancements in this field, a follow-up search, using with custom time-range filter, was conducted on January 12, 2025, in all databases that were searched earlier; the time range was from June 2023 until January 2025 to ensure our scoping review comprehensively captures evidence to date. Search strategy and inclusion criteria remained the same during the follow-up search process. The PRISMA-ScR flow diagram was updated with the new search results.

**Table 2.** Key words and Medical Subject Heading Descriptors

Descriptor	Key words	Medical Subject Heading term
Condition	Pregnancy-induced diabetes, gestational diabetes, gestational diabetes mellitus, GDM	Diabetes, gestational
Intervention	Probiotic*, probiotic supplement, lactobacillus, bifidobacterium	Probiotics
Study design	RCT	Randomized controlled trial

Abbreviations: GDM, gestational diabetes mellitus; RCT, randomized controlled trial.

**Table 3.** Search Strategy

Database	Search strategy	Results
PubMed	((((((Diabetes, Gestational[MeSH Terms]) OR (“Pregnancy-induced diabetes”)) OR (“gestational diabetes”)) OR (“gestational diabetes mellitus”)) OR (GDM)) AND ((((((((probiotics) AND (Probiotics[MeSH Terms])) OR (probiotic))) OR (lactobacillus[MeSH Terms])) OR (lactobacillus)) OR (bifidobacterium[MeSH Terms])) OR (bifidobacterium))) AND (((Randomized controlled trial[MeSH Terms]) OR (RCT)) OR (“clinical trial”)) Filters: Full text, Randomized controlled trial, in the last 10 years, English Sort by: Publication Date	28 + 3
Scopus	(TITLE-ABS-KEY (“gestational diabetes mellitus”)) OR (TITLE-ABS-KEY (“gestational diabetes”)) OR (TITLE-ABS-KEY (“pregnancy-induced diabetes”)) OR (TITLE-ABS-KEY (“pregnancy-induced diabetes”)) OR (TITLE-ABS-KEY (gdm)) AND (TITLE-ABS-KEY (probiotic*)) OR (TITLE-ABS-KEY (“probiotic supplement”)) OR (TITLE-ABS-KEY (lactobacillus)) OR (TITLE-ABS-KEY (bifidobacterium)) AND (TITLE-ABS-KEY (“Randomized controlled trial”)) OR (TITLE-ABS-KEY (rct)) AND (LIMIT-TO (LANGUAGE, “English”)) AND (LIMIT-TO (PUBYEAR, 2023) OR LIMIT-TO (PUBYEAR, 2022) OR LIMIT-TO (PUBYEAR, 2021) OR LIMIT-TO (PUBYEAR, 2020) OR LIMIT-TO (PUBYEAR, 2019) OR LIMIT-TO (PUBYEAR, 2018) OR LIMIT-TO (PUBYEAR, 2017) OR LIMIT-TO (PUBYEAR, 2016) OR LIMIT-TO (PUBYEAR, 2015) OR LIMIT-TO (PUBYEAR, 2014) OR LIMIT-TO (PUBYEAR, 2013))	110 + 10
Web of Science Core Collection	(((((((((ALL=(“Pregnancy-induced diabetes”)) OR ALL=(“gestational diabetes”)) OR ALL=(“gestational diabetes mellitus”)) OR ALL=(GDM)) AND ALL=(probiotic)) OR ALL=(“probiotic supplement”)) OR ALL=(lactobacillus)) OR ALL=(bifidobacterium)) AND ALL=(“Randomized controlled trial”)) OR ALL=(RCT) and Article (Document Types) and Obstetrics Gynecology or Nutrition Dietetics or Endocrinology Metabolism or Research Experimental Medicine or Biotechnology Applied Microbiology or Women’s Studies or Physiology or Cell Biology (Research Areas) and English (Languages) and Randomized controlled trial (Search within all fields) and Gdm (Search within all fields)	174 + 10
Cochrane Library	(MeSH descriptor (Diabetes, Gestational) explode all trees) OR “gestational diabetes mellitus” OR GDM AND (MeSH descriptor: [Probiotics] explode all trees) OR Probiotic* OR lactobacillus OR bifidobacterium AND (MeSH descriptor: [Randomized controlled trial] explode all trees) OR (Randomized controlled trial) OR RCT. Filters: Trial, publication date: 2013-2023, English.	44 + 14
Total		390

### Data Importing, Extracting, and Charting

Search results from electronic databases were downloaded as .ris and .nbib formatted files and were then imported into Rayyan, a semi-automated screening tool.^48^ First, the duplicates were removed using a built-in option in Rayyan. Then, blinding was turned on to ensure unbiased screening of all the included results after removing duplicates. Careful title and abstract screening of each study was carried out in consideration of inclusion and exclusion criteria by 2 reviewers independently, after which a full-text screening was done. To track, compile, and summarize the findings from the included RCTs that met the inclusion criteria, a custom template was developed in Microsoft Excel (Microsoft, Redmond, WA) to record the key characteristics of each RCT, including author name, publication year, country, study population, arms, intervention, comparator, major findings, and outcomes. Data were extracted by 1 reviewer (S.A.U.) from the included studies in MS Excel and was ratified by a co-author (A.T.).

### Statistical Analysis

Effect sizes (Cohen’s *d*) were calculated based on the available continuous data from all the RCTs for each outcome of interest reported as mean and SD or median and interquartile range,^49^ using the following formula: *d* = mean 1 – mean 2/pooled SD. The pooled SD calculation was based on the sample sizes of the study arms. Cohen’s *d* could not be calculated for parameters with no reported mean and SD or where the difference between the means was not statistically significant.

The following equations^49^ were used in in MS Excel, where the median and interquartile range were reported:


$$\begin{array}{c} \bar{\text{x}}\;\approx \;\left(q1+m+q3\right)/3\\ S\;\approx \;q3-q1/\left(2{ф}^{-1}\left(0.75n-0.125/\left(n+0.25\right)\right)\right) \end{array}$$

The SD was calculated from the CIs in studies where the SD was not reported with the mean. The R package esc_2x2 was used to calculate effect size (Hedges’ *g*) for categorical variables.^50^

## RESULTS

### Search Outcome

**Figure 1.**
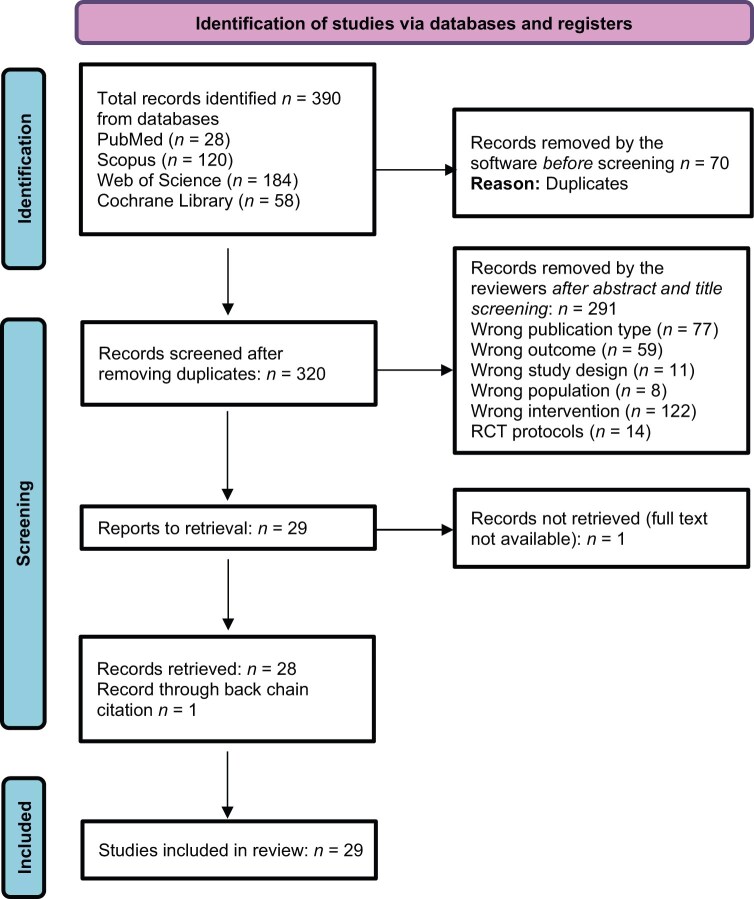
Preferred Reporting Items for Systematic Reviews and Meta-Analyses Extension for Scoping Reviews Flow Diagram. RCT, randomized controlled trial.

Searches in the electronic databases generated 390 RCTs that met the inclusion criteria. All search results were imported into Rayyan.^48^ The tool identified and removed 70 duplicate citations. Using a screening procedure based on titles and abstracts independently by 2 reviewers (S.A.U. and A.A.), 320 articles were screened. Of these, 291 articles that did not align with our research question were excluded for reasons such as “wrong study designs,” “wrong population,” “wrong outcome,” “wrong publication type,” and “wrong intervention.” These 291 articles also included 14 study protocols. Discordant results were resolved by discussion between the 2 reviewers. In the event of disagreement related to the inclusion or exclusion of the article between them, a third review (A.T.) was consulted. Twenty-nine studies could not be eliminated based on title and abstract and passed the eligibility for full-text assessment. One record could not be retrieved as a full-text article and was excluded. Reference lists of all the remaining 28 RCTs were checked using the back-chain citation method and 1 additional RCT meeting our eligibility criteria was identified. A total of 29 RCTs were then included in the review. Figure 1 illustrates the study selection process in a flow diagram, per the guidelines charted by PRISMA-ScR.

### Characteristics of the Included Studies

All included studies were double-blind, RCTs. Of all the studies included in the review, 3 RCTs were conducted in the Asia Pacific region (specifically, Australia^51^ and New Zealand^52^^,^^53^), and 5 RCTs were conducted in European countries (ie, Denmark,^54^ Ireland,^47^ and Finland^55–57^). Asia took the lead with 20 RCTs addressing this research question (conducted in Singapore,^58^ China,^59^ and Thailand^60^, 17 in Iran,^61–77^) and 1 trial was conducted in Israel.^78^ Nineteen studies were conducted in the past 5 years from 2018 to 2024,^51^^,^^52^^,^^54–60^^,^^62^^,^^64^^,^^65^^,^^68^^,^^70^^,^^73–76^^,^^78^ and the remaining 10 were conducted in the past 6 to 10 years from 2013 to 2017^47^^,^^53^^,^^61^^,^^63^^,^^66^^,^^67^^,^^69^^,^^71^^,^^72^^,^^77^ (Table 4).

**Table 4.** Characteristics of the Included Studies

Characteristic	No.	References
Location		
Asia Pacific	3	Callaway et al (2019)^51^
		Chen et al (2021)^52^
		Wickens et al (2017)^53^
Europe	5	Lindsay et al (2015)^47^
		Halkjaer et al (2020)^54^
		Houttu et al (2021)^55^
		Pellonperä et al (2019)^56^
		Mokkala et al (2022)^57^
Asia	20	Godfrey et al (2021)^58^
		Si et al (2019)^59^
		Kijmanawat et al (2019)^60^
		Ahmadi et al (2016)^61^
		Amirani et al (2022)^62^
		Asemi et al (2013)^63^
		Asgharian et al (2020)^64^
		Babadi et al (2019)^65^
		Badehnoosh et al (2018)^66^
		Dolatkhah et al (2015)^67^
		Hajifaraji et al (2018)^68^
		Jafarnejad et al (2016)^69^
		Jamilian et al (2019)^70^
		Karamali et al (2016)^71^
		Karamali et al (2018)^72^
		Nabhani et al (2022)^73^
		Nabhani et al (2018)^74^
		Sahhaf Ebrahimi et al (2019)^75^
		Shahriari et al (2021)^76^
		Jamilian et al (2016)^77^
Middle East	1	Nachum et al (2024)^78^
Year range		
2018-2024	19	Callaway et al (2019)^51^
		Chen et al (2021)^52^
		Halkjaer et al (2020)^54^
		Houttu et al (2021)^55^
		Pellonperä et al (2019)^56^
		Mokkala et al (2022)^57^
		Godfrey et al (2021)^58^
		Si et al (2019)^59^
		Kijmanawat et al (2019)^60^
		Amirani et al (2022)^62^
		Asgharian et al (2020)^64^
		Babadi et al (2019)^65^
		Hajifaraji et al (2018)^68^
		Jamilian et al (2019)^70^
		Nabhani et al (2022)^73^
		Nabhani et al (2018)^74^
		Sahhaf Ebrahimi et al (2019)^75^
		Shahriari et al (2021)^76^
		Nachum et al (2024)^78^
2013-2017	10	Lindsay et al (2015)^47^
		Wickens et al (2017)^53^
		Ahmadi et al (2016)^61^
		Asemi et al (2013)^63^
		Badehnoosh et al (2018)^66^
		Dolatkhah et al (2015)^67^
		Jafarnejad et al (2016)^69^
		Karamali et al (2016)^71^
		Karamali et al (2018)^72^
		Jamilian et al (2016)^77^
Study arms, no.		
2	25	Lindsay et al (2015)^47^
		Callaway et al (2019)^51^
		Chen et al (2021)^52^
		Wickens et al (2017)^53^
		Halkjaer et al (2020)^54^
		Godfrey et al (2021)^58^
		Si et al (2019)^59^
		Kijmanawat et al (2019)^60^
		Ahmadi et al (2016)^61^
		Amirani et al (2022)^62^
		Asemi et al (2013)^63^
		Asgharian et al (2020)^64^
		Babadi et al (2019)^65^
		Badehnoosh et al (2018)^66^
		Dolatkhah et al (2015)^67^
		Hajifaraji et al (2018)^68^
		Jafarnejad et al (2016)^69^
		Karamali et al (2016)^71^
		Karamali et al (2018)^72^
		Nabhani et al (2022)^73^
		Nabhani et al (2018)^74^
		Sahhaf Ebrahimi et al (2019)^75^
		Shahriari et al (2021)^76^
		Jamilian et al (2016)^77^
		Nachum et al (2024)^78^
3	1	Jamilian et al (2019)^70^
4	3	Houttu et al (2021)^55^
		Pellonperä et al (2019)^56^
		Mokkala et al (2022)^57^
Population		
Women aged >16 y	1	Wickens et al (2017)^53^
Women aged >18 y	5	Lindsay et al (2015)^47^
		Callaway et al (2019)^51^
		Halkjaer et al (2020)^54^
		Asgharian et al (2020)^64^
		Nachum et al (2024)^78^
Women aged 18-30 y	1	Asemi et al (2013)^63^
Women aged 18-37 y	1	Jamilian et al (2016)^77^
Women aged 18-38 y	1	Godfrey et al (2021)^58^
Women aged 18-40 y	6	Jamilian et al (2019)^70^
		Karamali et al (2016)^71^
		Karamali et al (2018)^72^
		Nabhani et al (2022)^73^
		Nabhani et al (2018)^74^
		Shahriari et al (2021)^76^
Women aged 18-45 y	4	Houttu et al (2021)^55^
		Mokkala et al (2022)^57^
		Kijmanawat et al (2019)^60^
		Hajifaraji et al (2018)^68^
No age criteria specified	4	Pellonperä et al (2019)^56^
		Si et al (2019)^59^
		Jafarnejad et al (2016)^69^
		Sahhaf Ebrahimi et al (2019)^75^
Pregnant	28	Lindsay et al (2015)^47^
		Callaway et al (2019)^51^
		Chen et al (2021)^52^
		Wickens et al (2017)^53^
		Halkjaer et al (2020)^54^
		Houttu et al (2021)^55^
		Pellonperä et al (2019)^56^
		Mokkala et al (2022)^57^
		Si et al (2019)^59^
		Kijmanawat et al (2019)^60^
		Ahmadi et al (2016)^61^
		Amirani et al (2022)^62^
		Asemi et al (2013)^63^
		Asgharian et al (2020)^64^
		Babadi et al (2019)^65^
		Badehnoosh et al (2018)^66^
		Dolatkhah et al (2015)^67^
		Hajifaraji et al (2018)^68^
		Jafarnejad et al (2016)^69^
		Jamilian et al (2019)^70^
		Karamali et al (2016)^71^
		Karamali et al (2018)^72^
		Nabhani et al (2022)^73^
		Nabhani et al (2018)^74^
		Sahhaf Ebrahimi et al (2019)^75^
		Shahriari et al (2021)^76^
		Jamilian et al (2016)^77^
		Nachum et al (2024)^78^
Planning to conceive in the next 6 mo	1	Godfrey et al (2021)^58^
Intervention		
Single-strain probiotics	4	Lindsay et al (2015)^47^
		Chen et al (2021)^52^
		Wickens et al (2017)^53^
		Si et al (2019)^59^
Multistrain probiotics	25	Callaway et al (2019)^51^
		Halkjaer et al (2020)^54^
		Houttu et al (2021)^55^
		Pellonperä et al (2019)^56^
		Mokkala et al (2022)^57^
		Godfrey et al (2021)^58^
		Kijmanawat et al (2019)^60^
		Ahmadi et al (2016)^61^
		Amirani et al (2022)^62^
		Asemi et al (2013)^63^
		Asgharian et al (2020)^64^
		Babadi et al (2019)^65^
		Badehnoosh et al (2018)^66^
		Dolatkhah et al (2015)^67^
		Hajifaraji et al (2018)^68^
		Jafarnejad et al (2016)^69^
		Jamilian et al (2019)^70^
		Karamali et al (2016)^71^
		Karamali et al (2018)^72^
		Nabhani et al (2022)^73^
		Nabhani et al (2018)^74^
		Sahhaf Ebrahimi et al (2019)^75^
		Shahriari et al (2021)^76^
		Jamilian et al (2016)^77^
		Nachum et al (2024)^78^
Probiotics vs placebo	15	Lindsay et al (2015)^47^
		Callaway et al (2019)^51^
		Chen et al (2021)^52^
		Wickens et al (2017)^53^
		Halkjaer et al (2020)^54^
		Kijmanawat et al (2019)^60^
		Babadi et al (2019)^65^
		Badehnoosh et al (2018)^66^
		Dolatkhah et al (2015)^67^
		Hajifaraji et al (2018)^68^
		Jafarnejad et al (2016)^69^
		Karamali et al (2016)^71^
		Shahriari et al (2021)^76^
		Jamilian et al (2016)^77^
		Nachum et al (2024)^78^
Probiotics + co-supplementation vs placebo	6	Houttu et al (2021)^55^
		Pellonperä et al (2019)^56^
		Mokkala et al (2022)^57^
		Godfrey et al (2021)^58^
		Amirani et al (2022)^62^
		Jamilian et al (2019)^70^
Synbiotics vs placebo	4	Ahmadi et al (2016)^61^
		Karamali et al (2016)^71^
		Nabhani et al (2022)^73^
		Nabhani et al (2018)^74^
Probiotic yogurt vs regular yogurt	3	Asemi et al (2013)^63^
		Asgharian et al (2020)^64^
		Sahhaf Ebrahimi et al (2019)^75^
Fermented black garlic vs regular black garlic	1	Si et al (2019)^59^
Outcome		
FPG	23	Lindsay et al (2015)^47^
		Callaway et al (2019)^51^
		Chen et al (2021)^52^
		Wickens et al (2017)^53^
		Halkjaer et al (2020)^54^
		Pellonperä et al (2019)^56^
		Godfrey et al (2021)^58^
		Si et al (2019)^59^
		Kijmanawat et al (2019)^60^
		Amirani et al (2022)^62^
		Asemi et al (2013)^63^
		Asgharian et al (2020)^64^
		Babadi et al (2019)^65^
		Badehnoosh et al (2018)^66^
		Dolatkhah et al (2015)^67^
		Jafarnejad et al (2016)^69^
		Jamilian et al (2019)^70^
		Karamali et al (2016)^71^
		Nabhani et al (2018)^74^
		Sahhaf Ebrahimi et al (2019)^75^
		Shahriari et al (2021)^76^
		Jamilian et al (2016)^77^
		Nachum et al (2024)^78^
1-h OGTT	9	Callaway et al (2019)^51^
		Chen et al (2021)^52^
		Wickens et al (2017)^53^
		Halkjaer et al (2020)^54^
		Pellonperä et al (2019)^56^
		Godfrey et al (2021)^58^
		Si et al (2019)^59^
		Asgharian et al (2020)^64^
		Shahriari et al (2021)^76^
2-h OGTT	9	Callaway et al (2019)^51^
		Chen et al (2021)^52^
		Wickens et al (2017)^53^
		Halkjaer et al (2020)^54^
		Pellonperä et al (2019)^56^
		Godfrey et al (2021)^58^
		Si et al (2019)^59^
		Asgharian et al (2020)^64^
		Shahriari et al (2021)^76^
Serum insulin	10	Lindsay et al (2015)^47^
		Chen et al (2021)^52^
		Pellonperä et al (2019)^56^
		Kijmanawat et al (2019)^60^
		Dolatkhah et al (2015)^67^
		Jafarnejad et al (2016)^69^
		Jamilian et al (2019)^70^
		Karamali et al (2016)^71^
		Nabhani et al (2018)^74^
		Jamilian et al (2016)^77^
HOMA-IR	9	Lindsay et al (2015)^47^
		Chen et al (2021)^52^
		Kijmanawat et al (2019)^60^
		Dolatkhah et al (2015)^67^
		Jafarnejad et al (2016)^69^
		Jamilian et al (2019)^70^
		Karamali et al (2016)^71^
		Nabhani et al (2018)^74^
		Jamilian et al (2016)^77^
HOMA2-IR	1	Pellonperä et al (2019)^56^
HOMA-β	3	Ahmadi et al (2016)^61^
		Karamali et al (2016)^71^
		Jamilian et al (2016)^77^
QUICKI	8	Ahmadi et al (2016)^61^
		Amirani et al (2022)^62^
		Babadi et al (2019)^65^
		Dolatkhah et al (2015)^67^
		Jamilian et al (2019)^70^
		Karamali et al (2016)^71^
		Nabhani et al (2018)^74^
		Jamilian et al (2016)^77^
Serum hs-CRP	7	Houttu et al (2021)^55^
		Badehnoosh et al (2018)^66^
		Hajifaraji et al (2018)^68^
		Jafarnejad et al (2016)^69^
		Jamilian et al (2019)^70^
		Karamali et al (2018)^72^
		Nabhani et al (2022)^73^
		Jamilian et al (2016)^77^
C-peptide	1	Lindsay et al (2015)^47^
HbA_1c_	2	Jafarnejad et al (2016)^69^
		Sahhaf Ebrahimi et al (2019)^75^
Postprandial glucose	1	Sahhaf Ebrahimi et al (2019)^75^
CatD	1	Mokkala et al (2022)^57^
IL-1	1	Babadi et al (2019)^65^
IL-6	2	Hajifaraji et al (2018)^68^
		Jafarnejad et al (2016)^69^
IL-8	1	Babadi et al (2019)^65^
IL-10	1	Jafarnejad et al (2016)^69^
TNF-α	3	Babadi et al (2019)^65^
		Hajifaraji et al (2018)^68^
		Jafarnejad et al (2016)^69^
IFN-γ	1	Jafarnejad et al (2016)^69^
MMP-8	1	Houttu et al (2021)^55^
IGFBP-1	1	Houttu et al (2021)^55^
phIGFBP-1	1	Houttu et al (2021)^55^
TGF-β	1	Babadi et al (2019)^65^
VEGF	1	Babadi et al (2019)^65^
Incidence of GDM	6	Callaway et al (2019)^51^
		Chen et al (2021)^52^
		Wickens et al (2017)^53^
		Halkjaer et al (2020)^54^
		Godfrey et al (2021)^58^
		Shahriari et al (2021)^76^

Abbreviations: CatD, cathepsin D; FPG, fasting plasma glucose; GDM, gestational diabetes mellitus; HOMA, homeostatic model assessment; HOMA2, updated homeostatic model assessment; hs-CRP, high-sensitivity C-reactive protein; IFN, interferon; IGFBP-1, insulin-like growth factor binding protein-1; IL, interleukin; IR, insulin resistance; MMP-8, matrix metalloproteinase-8; NS, not significant; OGTT, oral glucose tolerance test; phIGFBP-1, phosphorylated insulin-like growth factor binding protein-1; QUICKI, quantitative insulin sensitivity check index; TNF, tumor necrosis factor; VEGF, vascular endothelial growth factor.

Twenty-five of 29 RCTs had 2 study arms.^47^^,^^51–54^^,^^58–69^^,^^71–78^ One RCT was 3-armed^70^ and the remaining 3 RCTs were 4-armed.^55–57^ All studies recruited pregnant women,^47^^,^^51–57^^,^^59–78^ except 1,^58^ for which the participants were recruited if they planned to conceive in the next 6 months. One study^53^ recruited women if they were aged 16 years or older, and 5^47^^,^^51^^,^^54^^,^^64^^,^^78^ recruited women if they were aged 18 years or older. None of these specified the maximum age limit for recruitment. Thirteen RCTs^55^^,^^57^^,^^58^^,^^60^^,^^63^^,^^68^^,^^70–74^^,^^76^^,^^77^ recruited participants aged between 18 and 45 years; 4 studies^56^^,^^59^^,^^69^^,^^75^ did not mention any age criteria (Table 4).

Only 4 of 29 RCTs used single-strain probiotics as an intervention^47^^,^^52^^,^^53^^,^^59^ and the remaining 25 RCTs had multistrain probiotics.^51^^,^^54–58^^,^^60–78^ The probiotics used in the intervention of these RCTs included *Lactobacillus acidophilus, Lacticaseibacillus casei, Lactobacillus delbrueckii, Limosilactobacillus fermentum* (formerly *Lactobacillus fermentum*), *Lacticaseibacillus rhamnosus (*formerly *Lactobacillus rhamnosus*), *Lactobacillus gasseri, L. bulgaricus, Lactiplantibacillus plantarum (*formerly *Lactobacillus plantarum), Lacticaseibacillus paracasei* (formerly *Lactobacillus paracasei*), *Limosilactobacillus reuteri (*formerly *Lactobacillus reuteri*), *Ligilactobacillus salivarius (*formerly *Lactobacillus salivarius*), *Bifidobacterium bifidum*, *B. lactis*, *B. longum*, *B. animalis* ssp. *lactis*, *B. breve*, *B. infantis*, and *Streptococcus thermophilus*. Randomized controlled trials included in the review, including those published after 2020, used the traditional bacterial nomenclature for probiotic species such as *Lactobacillus* and *Bifidobacterium*. However, following the updated taxonomic guidelines and reclassification by Zheng et al^79^ for *Lactobacillus*, we attempted to use the current nomenclature in accordance with the International Code of Nomenclature and the recommendations by the ISAAP. Where applicable, updated genus names were used in the review, while we recognized the included trials may use the former nomenclature.

The interventions in these RCTs lasted a minimum of 4 weeks and continued for up to 6 months postpartum. The probiotic dose varied among all studies. Capsules were used as vehicles in 25 studies^47^^,^^51–58^^,^^60–62^^,^^65–74^^,^^76–78^; probiotics were given as supplements either alone or in combination with micronutrients in these studies, whereas 4 studies^59^^,^^63^^,^^64^^,^^75^ used food (yogurt and garlic) to introduce probiotics as an intervention (Table 4).

Of the total 29 RCTs, 15^47^^,^^51–54^^,^^60^^,^^65–69^^,^^71^^,^^76–78^ compared probiotic supplement with a placebo and 6 RCTs used probiotics co-supplemented with micronutrients and compared the effect with that of placebo.^55–58^^,^^62^^,^^70^ Four studies used synbiotics and placebo,^61^^,^^72–74^ and 3 trials ascertained the comparative effects of probiotic yogurt versus regular yogurt.^63^^,^^64^^,^^75^ One study identified the impact of fermented black garlic in contrast to the regular black garlic effect^59^ (Table 4).

### Measured Clinical Outcomes

The clinical parameters measured in these RCTs that related to glucose metabolism included serum insulin, HOMA-IR, the updated HOMA-IR (HOMA2-IR), HOMA-β, QUICKI, FPG, HbA_1c_, 1-h OGTT, 2-h OGTT, and incidence of GDM.^47^^,^^51–54^^,^^56^^,^^58–67^^,^^69–71^^,^^74–78^ TNF-α, IFN-γ, C-peptide, serum cathepsin D (CatD), IL-1, IL-6, IL-8, IL-10, TGF-β, VEGF, hs-CRP, matrix metalloproteinase-8 (MMP-8), phIGFBP-1, and IGFBP-1 were reported to measure inflammation^47^^,^^55^^,^^57^^,^^65^^,^^66^^,^^68–70^^,^^72^^,^^73^^,^^77^ (Table 4).

### Effect Size

Cohen’s *d* values were reported from continuous and categorical data and categorized as very large (>0.8), large (≤0.8), medium (≤0.5), or small (≤0.2) effect sizes. Clinical parameters related to glucose metabolism mostly had medium to large and very large effect sizes. The effect size for the incidence of GDM was medium to large. Table 5 summarizes the effect sizes of all the parameters.

**Table 5.** Summary of the effects of probiotic supplementation on glucose and inflammatory biomarkers

Clinical parameter	Effect	*P* value	Cohen’s *d* value	Effect size	Reference
Fasting plasma glucose	NS	NA	NA	NA	Lindsay et al (2015)^47^
					Halkjaer et al (2020)^54^
					Pellonperä et al (2019)^56^
					Godfrey et al (2021)^58^
					Asemi et al (2013)^63^
					Jafarnejad et al (2016)^69^
					Nabhani et al (2018)^74^
					Shahriari et al (2021)^76^
					Jamilian et al (2016)^77^
					Nachum et al (2024)^78^
	Decreased	0.048	0.05	Small	Wickens et al (2017)^53^
		0.040	0.10^a^	Small	Chen et al (2021)^52^
		0.049	0.21	Medium	Callaway et al (2019)^51^
		0.02	0.24	Medium	Babadi et al (2019)^65^
		0.048	0.31	Medium	Sahhaf Ebrahimi et al (2019)^75^
		0.008	0.33	Medium	Asgharian et al (2020)^64^
		0.001	0.55 Ϯ	Large	Si et al (2019)^59^
		0.03	0.57	Large	Kijmanawat et al (2019)^60^
		0.004	0.65	Large	Amirani et al (2022)^62^
		0.01	0.67	Large	Badehnoosh et al (2018)^66^
		0.001	0.84	Very large	Jamilian et al (2019)^70^
		0.001	0.95	Very large	Karamali et al (2016)^71^
		0.02	3.20	Very large	Dolatkhah et al (2015)^67^
1-h OGTT	NS	NA	NA	NA	Callaway et al (2019)^51^
					Chen et al (2021)^52^
					Wickens et al (2017)^53^
					Halkjaer et al (2020)^54^
					Pellonperä et al (2019)^56^
					Godfrey et al (2021)^58^
					Asgharian et al (2020)^64^
					Shahriari et al (2021)^76^
	Decreased	0.006	0.42^b^	Medium	Si et al (2019)^59^
2-h OGTT	NS	NA	NA	NA	Callaway et al (2019)^51^
					Chen et al (2021)^52^
					Wickens et al (2017)^53^
					Halkjaer et al (2020)^54^
					Pellonperä et al (2019)^56^
					Shahriari et al (2021)^76^
	Decreased	0.02	0.16^c^	Small	Godfrey et al (2021)^58^
		0.002	0.49	Medium	Asgharian et al (2020)^64^
		0.001	0.56^b^	Large	Si et al (2019)^59^
Serum insulin	NS	NA	NA	NA	Lindsay et al (2015)^47^
					Chen et al (2021)^52^
					Pellonperä et al (2019)^56^
					Dolatkhah et al (2015)^67^
					Nabhani et al (2018)^74^
	Decreased	0.01	0.33	Medium	Jamilian et al (2019)^70^
		0.03	0.56	Large	Jamilian et al (2016)^77^
		0.01	0.68	Large	Karamali et al (2016)^71^
		0.04	1.15	Very large	Jafarnejad et al (2016)^69^
		0.01	1.56	Very large	Kijmanawat et al (2019)^60^
HOMA-IR	NS	NA	NA	NA	Lindsay et al (2015)^47^
					Chen et al (2021)^52^
					Nabhani et al (2018)^74^
	Decreased	0.001	0.50	Medium	Jamilian et al (2019)^70^
		0.04	0.60	Large	Jamilian et al (2016)^77^
		0.001	0.80	Large	Karamali et al (2016)^71^
		0.03	0.84	Very large	Jafarnejad et al (2016)^69^
		0.01	1.53	Very large	Kijmanawat et al (2019)^60^
		0.03	3.27	Very large	Dolatkhah et al (2015)^67^
HOMA2-IR	NS	NA	NA	NA	Pellonperä et al (2019)^56^
HOMA-β	NS	NA	NA	NA	Karamali et al (2016)^71^
	Decreased	0.03	0.55	Large	Jamilian et al (2016)^77^
		0.008	0.66	Large	Ahmadi et al (2016)^61^
QUICKI	NS	NA	NA	NA	Dolatkhah et al (2015)^67^
					Nabhani et al (2018)^74^
	Increased	0.001	0.00	Small	Babadi et al (2019)^65^
		0.03	0.53	Large	Jamilian et al (2016)^77^
		0.05	0.55	Large	Jamilian et al (2019)^70^
		0.002	0.92	Very large	Amirani et al (2022)^62^
		0.007	1.08	Very large	Karamali et al (2016)^71^
		0.02	1.08	Very large	Ahmadi et al (2016)^61^
HbA_1c_	NS	NA	NA	NA	Jafarnejad et al (2016)^69^
	Decreased	0.025	0.33	Medium	Sahhaf Ebrahimi et al (2019)^75^
Postprandial glucose	NS	NA	NA	NA	Nachum et al (2024)^78^
	Decreased	0.002	0.74	Large	Sahhaf Ebrahimi et al (2019)^75^
Incidence of GDM	NS	NA	NA	NA	Callaway et al (2019)^51^
					Halkjaer et al (2020)^54^
					Godfrey et al (2021)^58^
					Shahriari et al (2021)^76^
	Decreased	0.047	0.39 Ϯ	Medium	Chen et al (2021)^52^
		0.03	0.66 Ϯ	Large	Wickens et al (2017)^53^
Serum hs-CRP	NS	NA	NA	NA	Halkjaer et al (2020)^54^
					Houttu et al (2021)^55^
					Nabhani et al (2022)^73^
	Decreased	0.001	0.72	Large	Jamilian et al (2019)^70^
		0.004	0.76	Large	Jamilian et al (2016)^77^
		0.002	0.78	Large	Karamali et al (2018)^72^
		0.03	1.60	Very large	Jafarnejad et al (2016)^69^
C-peptide	NS	NA	NA	NA	Lindsay et al (2015)^47^
CatD	NS	NA	NA	NA	Mokkala et al (2022)^57^
IL-1	NS	NA	NA	NA	Babadi et al (2019)^65^
IL-6	NS	NA	NA	NA	Hajifaraji et al (2018)^68^
	Decreased	0.04	1.66	Very large	Jafarnejad et al (2016)^69^
IL-8	NS	NA	NA	NA	Babadi et al (2019)^65^
IL-10	NS	NA	NA	NA	Jafarnejad et al (2016)^69^
TNF-α	NS	NA	NA	NA	Hajifaraji et al (2018)^68^
	Decreased	0.03	^d^	^d^	Babadi et al (2019)^65^
		0.04	1.18	Very large	Jafarnejad et al (2016)^69^
IFN-γ	NS	NA	NA	NA	Jafarnejad et al (2016)^69^
MMP-8	NS	NA	NA	NA	Houttu et al (2021)^55^
IGFBP-1	NS	NA	NA	NA	Houttu et al (2021)^55^
phIGFBP-1	NS	NA	NA	NA	Houttu et al (2021)^55^
TGF-β	Increased	0.002	^d^	^d^	Babadi et al (2019)^65^
VEGF	Increased	0.006	^d^	^d^	Babadi et al (2019)^65^

a Mean and SD values are not reported.

b Effect size based on RR in R.^50^

c Effect size calculated with median and interquartile range.^49^

d Approximated.

Abbreviations: CatD, cathepsin D; GDM, gestational diabetes mellitus; HOMA, homeostatic model assessment; HOMA2, updated homeostatic model assessment; hs-CRP, high-sensitivity C-reactive protein; IFN, interferon; IGFBP-1, insulin-like growth factor binding protein-1; IL, interleukin; IR, insulin resistance; MMP-8, matrix metalloproteinase-8; NA, not applicable; NS, not significant; OGTT, oral glucose tolerance test; phIGFBP-1, phosphorylated insulin-like growth factor binding protein-1; QUICKI, quantitative insulin sensitivity check index; TGF, transforming growth factor; TNF, tumor necrosis factor; VEGF, vascular endothelial growth factor.

### Probiotics’ Effect on Glucose Metabolism

Evidence from the included trials reveals mixed results about the probiotics’ effect on glucose metabolism. Ten^47^^,^^54^^,^^56^^,^^58^^,^^63^^,^^69^^,^^74^^,^^76–78^ of 23 studies did not find any significant differences between placebo and intervention group for FPG. However, 13 RCTs^51–53^^,^^59^^,^^60^^,^^62^^,^^64–67^^,^^70^^,^^71^^,^^75^ reported a significant decrease in FPG level in the probiotic group compared with placebo. The magnitude of the effect in the probiotics group was either medium, large, or exceptionally large, except for 2 studies^52^^,^^53^ belonging to the same trial with different objectives and published separately, in which the effect sizes were measured to be small. These 2 studies used a single-strain probiotic at the dose of 6 × 10^9^ CFU daily of *Lacticaseibacillus rhamnosus* HN001 in a capsule form from 14 to 16 weeks of gestation until 6 months postpartum. Four studies^51^^,^^64^^,^^65^^,^^75^ reported medium effect sizes, with 1 study administering probiotic yogurt containing additional *Lactobacillus acidophilus* LA-5 and *B. lactis* BB-12 from 24 weeks of gestation until delivery. The remaining 3 studies administered capsules of multistrain probiotics containing strains of *Lactobacillus* and *Bifidobacterium* in doses from 10^6^ up to 2 × 10^9^ CFU each strain per gram of capsule with varying periods from as short as 6 weeks to as long as 20-24 weeks in case of full-term. Randomized controlled trials demonstrating large^59^^,^^60^^,^^62^^,^^66^ and very large effect sizes^67^^,^^70^^,^^71^ administered multi-strain probiotic capsules containing no less than 10^9^ CFU per strain over relatively short durations of 4-8 weeks (Table 5).

Nine studies measured 1- and 2-hour OGTT. For 1-hour OGTT, 8 of 9 studies^51–54^^,^^56^^,^^58^^,^^64^^,^^76^ reported that probiotic supplementation did not significantly decrease 1-hour OGTT values compared with placebo. Only 1 study^59^ with a medium-sized effect reported a significant difference in the 1-hour OGTT values between the intervention and placebo groups and used 5 g of black garlic fermented with *L. bulgaricus* containing 10^8^ CFU mL^–1^ distilled water consumed daily for the entire duration of pregnancy. Comparable results with no significant differences between the probiotic and placebo group for 2-hour OGTT were achieved, except for 3 studies^58^^,^^59^^,^^64^ with varying effect sizes. All 3 studies had a longer duration of intervention and used different probiotics. A moderate to strong effect was observed by administering single-strain probiotic *L. bulgaricus* as an intervention (10^8^ CFU mL^–1^ distilled water per day) for 40 weeks,^59^ whereas the study with a medium-sized effect used multistrain probiotics including *Streptococcus thermophilus* and *L. delbrueckii* subsp. *bulgaricus* (10^7^ CFU g^–1^ each) in addition to *L. acidophilus* LA-5 and *B. lactis* BB-12 (5 × 10^8^ CFU g^–1^ each).^64^ A smaller effect size was observed in the study in which the probiotic intervention included *L. rhamnosus* NCC 4007 and *B. animalis* ssp*. lactis* NCC 2818 (10^10^ CFU d^–1^)^58^ (Table 5).

There is varying evidence regarding the effect of probiotic intervention on serum insulin, HOMA-IR, and QUICKI. Probiotic intervention did not affect serum insulin levels differently from placebo in 5 of 10 studies^47^^,^^52^^,^^56^^,^^67^^,^^74^ that measured serum insulin levels. Similarly, 9 studies measured HOMA-IR, of which 3^47^^,^^52^^,^^74^ did not report any significant effect of probiotic intervention on HOMA-IR levels. Two^67^^,^^74^ of 8 studies also reported that probiotic intervention did not affect QUICKI values of pregnant GDM women. However, Jafarnejad et al^69^ reported a significant decrease in serum insulin and HOMA-IR, demonstrating a considerable effect size. The intervention included a probiotic capsule containing 8 strains of probiotics (*S. thermophilus*, *B. breve*, *B. longum*, *B. infantis*, *L. acidophilus*, *Lactiplantibacillus plantarum*, *Lactobacillus paracasei*, and *L. delbrueckii* subsp. *bulgaricus*) in doses as high as 112.58 × 10^9^ CFU capsule^–1^, with 2 capsules to be consumed per day for 8 weeks. Nevertheless, this probiotic intervention did not significantly affect the HbA_1c_ level of the study participants.^69^ Jamilian et al^70^ also administered multispecies probiotics with 4 strains (*B. bifidum*, *L. acidophilus*, *Limosilactobacillus reuteri*, and *L. fermentum*) in a total dose of 8 × 10^9^ CFU d^–1^ for 6 weeks and observed a significant decrease in serum insulin level, HOMA-IR, and increase in QUICKI with medium and large effect sizes. Karamali et al^71^ reported similar findings that a multistrain probiotic intervention containing *L. acidophilus*, *L. casei*, and *B. bifidum* in doses of 2 × 10^9^ CFU g^–1^ each for 6 weeks significantly decreased serum insulin levels and HOMA-IR and increased QUICKI. The study demonstrated notable effect sizes between the intervention and control groups. However, this intervention did not affect HOMA2-IR. An RCT conducted in 2016 by Jamilian et al^77^ used exactly similar encapsulated probiotic intervention containing *L. acidophilus*, *L. casei*, and *B. bifidum* with the same doses (2 × 10^9^ CFU g^–1^ each strain), with consumption of 1 capsule day^–1^ and reported a significant decrease in serum insulin level, HOMA-IR, and HOMA-β, and an increase in QUICKI, exhibiting strong intervention effects. Another study used only 2 strains (*L. acidophilus* and *B. bifidum*) in capsule form with doses of 10^9^ CFU for each strain to be consumed for 4 weeks, which significantly reduced serum insulin levels and HOMA-IR, demonstrating a pronounced effect size of the intervention.^60^ The impact of the probiotic intervention on improved HOMA-IR in women with GDM was further confirmed by an 8-week intervention study^67^ in which encapsulated bacterial strains including *L. acidophilus* LA-5, *B. animalis* subsp. *lactis* BB-12, *Streptococcus thermophilus* STY-31, and *L. delbrueckii* subsp. *bulgaricus* LBY-27 were administered in the total dose of 4 × 10^9^ CFU, resulting in significant improvement. Findings from 2 RCTs^61^^,^^62^ reaffirmed the impact of short-term (6 weeks) multistrain probiotic intervention containing *L. acidophilus*, *L. casei*, *B. bifidum*, *B. lactis*, and *B. longum* on glucose metabolism by reducing HOMA-β and improving values of QUICKI in women with GDM with appreciable effect sizes. Both studies used the same dose of 2 × 10^9^ CFU g^–1^ each in co-supplementation with inositol or selenium daily. Only 1 RCT^65^ reported a small effect size and significantly increased QUICKI in women with GDM. The intervention in this study used a multistrain probiotic capsule containing *L. acidophilus*, *L. casei*, *B. bifidum*, and *Limosilactobacillus fermentum* in a dosage of 2 × 10^9^ CFU g^–1^ each strain every day for 6 weeks.

Sahhaf Ebrahimi et al^75^ measured HbA_1c_ and postprandial glucose level after an intervention with fermented yogurt containing additional probiotic strains of *B. lactis* and *L. acidophilus* in addition to *S. thermophilus* and *L. bulgaricus*, which are present in conventional yogurt. An 8-week consumption of 300 g of probiotic yogurt each day in high-risk women with GDM improved HbA_1c_ and postprandial glucose with moderate to strong effect sizes, respectively. Six studies measured the incidence of GDM, and only 2 studies^52^^,^^53^ reported a decrease with encapsulated probiotic intervention; the remaining 4 studies^51^^,^^54^^,^^58^^,^^76^ reported an insignificant effect. Both studies^52^^,^^53^ that reported positive effects were part of the same trial and used a single-strain encapsulated probiotic containing *L. rhamnosus* HN001 at the dose of 6 × 10^9^ per day. Both studies were published separately in different years.

### Probiotics and Inflammation

There has been limited research on probiotics’ role in improving inflammatory biomarkers in women with GDM. Only a few RCTs have investigated the use of probiotics during pregnancy. A short-duration 8-week trial^69^ with freeze-dried, encapsulated, multistrain probiotic intervention containing 8 various lactic acid bacterial strains (*S. thermophilus*, *B. breve*, *B. longum*, *B. infantis*, *L. acidophilus*, *Lactiplantibacillus plantarum*, *L. paracasei*, and *Lactobacillus delbrueckii* subsp. *bulgaricus*) in a dose of 112.5 × 10^9^ CFU per capsule per day decreased serum hs-CRP, IL-6, and TNF-α levels. The difference between the intervention and control arms was significant, with a considerable effect size. In the same study, IL-10 and IFN-γ concentrations increased and decreased, respectively, in the probiotic group from baseline to the eighth week of supplementation, but the change compared with the control group was not statistically significant. Jamalian et al^70^ and Karamali et al^72^ also explored the impact of multistrain probiotic interventions (*L. acidophilus*, *Limosilactobacillus reuteri*, *L. fermentum*, *B. bifidum*, and *Lactobacllus acidophilus, L. caseii*, and *B. bifidum*, respectively) on inflammation during gestation in women with GDM in addition to either vitamin D or inulin, respectively, as co-supplementation for 6 weeks. Both trials reported a significant decrease in hs-CRP levels with a pronounced effect. Similar results were achieved in another RCT^77^ in which the intervention was not co-supplemented and included only multistrain encapsulated probiotics (*L. acidophilus*, *L. casei*, and *B. bifidum*; 2 × 10^9^ CFU g^–1^ each with 1 capsule consumption per day). The serum hs-CRP level decreased significantly with a large effect size after 12 weeks of intervention in the group receiving the probiotic intervention compared with the placebo group. However, 3^54^^,^^55^^,^^73^ of 7 studies did not report any significant impact of the probiotic intervention on hs-CRP values. All 3 studies were conducted in different world regions and used multistrain probiotics despite varying higher doses of probiotics (ranging from a total of 20 billion CFU d^–1^ to 450 billion CFU d^–1^). Five more RCTs^47^^,^^55^^,^^57^^,^^65^^,^^68^ did not observe any significant reduction in inflammatory biomarkers such as C-peptide, IL-1, IL-6, IL-8, TNF-α, and CatD. However, the gene expression of TNF-α was downregulated with the upregulated gene expression of TGF-β and VEGF in individuals consuming multispecies probiotic supplements containing *L. acidophilus*, *L. casei*, *Limosilactobacillus fermentum*, and *B. bifidum* (2 × 10^9^ CFU g^–1^ each strain) for 6 weeks.^65^ Effect sizes could not be calculated for these parameters, due to the lack of data on means and SD.

## DISCUSSION

In this scoping review, we aimed to elucidate and critically review the role of probiotics in the management of GDM by improving glucose metabolism and reducing inflammation, focusing on *Lactobacillus* and *Bifidobacterium* strains as the main probiotics tested in clinical trials. To achieve our objectives, we mapped 29 RCTs that assessed the effect of *Lactobacillus* and *Bifidobacterium* species in the therapeutic management of GDM, published between 2013 and 2025. In essence, all included trials in this review concluded that probiotics intervention is promising in managing GDM.

Probiotics are live nonpathogenic microorganisms that improve dysbiosis and increase the expression of adhesion proteins within the intestinal epithelium, which reduces intestinal permeability and insulin resistance.^31^ Each probiotic genus and strain exerts different effects on metabolic and inflammatory pathways depending on the initial dose, strain, quality, and storage conditions. The strains and doses thereof administered in the included trials varied because no standard guidelines on probiotic dosage for pregnant women have been set until now. Although strains were wide ranging, almost all of them belonged to the Bifidobacteriaceae or Lactobacillaceae families owing to an increased interest of the scientific community in their ability to improve gut dysbiosis in addition to their efficacy in the treatment of other gastrointestinal pathologies.^80^ The choice of these 2 genera among all others in the included trials can be attributed to their safety profile, which takes into consideration the dose, duration, frequency of administration, the extent to which they might pose a threat to the consumer, and last, the nature of microbes themselves. However, the microbes used as probiotics are generally nonpathogenic, and the risk posed to health is nearly negligible. For instance, probiotic consumption over 100 years in France has led to only 1 case of *Lactobacillus* infection per 100 million people.^81^ Moreover, the risk of bacteremia after consumption of a *Lactobacillus* strain was reported to be fewer than 1 case per million consumers.^39^ There have been no documented reports of infections associated with the use of probiotics containing *Bifibacterium* species.^82^ Further evidence regarding the safety of these probiotics suggests their use in patients with compromised health status like those with Crohn’s disease, premature babies, or the elderly.^83^^,^^84^ In all the trials reviewed, there were no reports of bacterial infection among pregnant women despite doses as high as 450 billion CFU d^–1^. The paucity of opportunistic pathogenicity of these probiotics has further been confirmed by a review article covering a population of more than 1500 pregnant women, in whom the probiotic doses administered in the last trimester did not affect pregnancy outcomes.^85^ This finding is consistent with another study, which reported that supplementation with *Lactobacillus* and *Bifidobacterium* species during pregnancy does not pose any risk of adverse effects.^86^ However, evidence has also prompted exercising caution with probiotic use during pregnancy because there is an increased risk of preeclampsia but not of other adverse pregnancy outcomes.^87^

Our scoping review indicated striking results regarding the effect of *Lactobacillus*- and *Bifidobacterium*-based probiotics on glucose metabolism, with the majority of studies confirming that *Lactobacillus* and *Bifidobacterium* supplementation helps reduce FPG, 1-hour, and 2-hour OGTT in women with GDM. The results have been confirmed in other studies that used both human and animal models with diverse populations. Similar results were achieved when a probiotic yogurt containing *L. acidophilus* LA-5 and *B. lactis* BB-12, in addition to *L. bulgaricus* and *S. thermophilus,* was administered for 6 weeks to people with T2DM.^88^ Consuming probiotic fermented milk containing *L. acidophilus* LA-5 and *B. animalis* subsp*. lactis* BB-12 (10^9^ CFU d^–1^, each) for 6 weeks improved glycemic control in people with T2DM.^89^ Similarly, pooled results of RCTs studying probiotics as promising agents for women with GDM also concluded that probiotic yogurt led to a significant reduction in FPG (Hedges’ *g* = –0.37; 95% CI, –0.68 to –0.05; *I*^2^ = 0.0%).^90^ The results of the animal model–based research also corroborate our conclusions. In diabetic mice, the administration of *L. casei*^91^ and *L. acidophilus*^92^ reduced plasma glucose concentrations. Likewise, *Latilactobacillus sakei* (formerly *Lactobacillus sakei*) OK67, isolated from kimchi, ameliorated high-fat-diet–induced hyperglycemia in diabetic mice by suppressing the production of LPS, thereby reducing inflammation and enhancing the gene expression of occludin, claudin-1, and ZO-1 after 4.5 weeks of administration.^93^ On the other hand, there have been some contrasting findings concerning the effect of probiotics on glucose metabolism.^87^ Zheng et al concluded that *Lactobacillus* and *Bifidobacterium* supplementation does not significantly reduce FPG levels in GDM.^32^

Different formulations of probiotics may have varying effects. In this review, we included studies testing both food and formulated preparations of probiotics. Three trials used probiotic yogurt as an intervention. Two^64^^,^^75^ of 3 reported medium effect sizes (0.31 and 0.33 as absolute values) to determine the magnitude of probiotic yogurt effect on FPG. The used probiotics were *L. casei*, *Limo. fermentum*, *L. acidophilus*, *L. bulgaricus*, *B. bifidum*, *B. lactis*, *B. animalis*, and *S. thermophilus*. Similar results were obtained with the administration of encapsulated probiotics containing *Ligi. salivarius*, *L. acidophilus*, *L. casei*, *L. rhamnosus*, *B. animalis*, *B. lactis*, *B. longum*, *B. breve*, and *B. infantis* on FPG.^65^^,^^78^ In addition to this, an RCT investigating the effect of multispecies probiotics formulated in a capsule containing 3 freeze-dried strains, *B. longum* (2 × 10^7^ CFU capsule^–1^), *L*. *bulgaricus* (2 × 10^6^ CFU capsule^–1^) and *S. thermophilus* (2 × 10^6^ CFU capsule^–1^), with 3 capsules to be consumed for 3 months along with insulin revealed that the regimen significantly reduced fasting glucose levels and 30-minute postprandial glucose in men with type 1 diabetes mellitus.^94^ These findings align with evidence that both food and commercial preparations of probiotics may serve as an effective adjunct in conventional diabetes management.^94^ The majority of the studies demonstrating the beneficial effects of *Lactobacillus*- and *Bifidobacterium*-based probiotics have been of short-term duration.

Concerning the measured clinical outcomes, most of the included studies reporting serum insulin, HOMA-IR, and QUICKI demonstrated notable positive improvements in these parameters with administration of *Lactobacillus* and *Bifidobacterium* strains. HbA_1c_, being the gold standard for monitoring blood glucose levels,^95^ did not significantly improve with probiotics supplementation containing *Lactobacillus* and *Bifidobacterium* strains in women with GDM^96^^,^^97^; however, an improvement was observed in HbA_1c_ in patients with T2DM with the same supplementation.^96^

The significance of the probiotic intervention toward reduced incidence of GDM is still under scrutiny. Some clinical trials in our review indicated a substantial association between probiotic intake and the reduced incidence of GDM with notable intervention effects (medium to large), whereas others found no association at all. This beneficial link was further validated by Laitinen et al^30^ who concluded that dietary counselling and probiotic supplementation containing *L. rhamnosus* and *B. lactis* administered to normoglycemic women during pregnancy resulted in reduced risk of elevated glucose concentration, decreased serum insulin, HOMA, and increased QUICKI compared to placebo groups during the trimester of pregnancy.^16^ The incidence of GDM was reduced by 33% with *Lactobacillus* and *Bifidobacterium* supplementation; a more pronounced effect was observed with multistrain probiotics in women with GDM.^98^

Research studies have demonstrated possible metabolic pathways that support explanations of how *Lactobacillus* and *Bifidobacterium* improve glucose metabolism. The probiotic-derived SCFAs include acetate, propionate, and butyrate, which constitute around 95% of all SCFAs, with acetate being in the predominant concentration. These fatty acids bind to G-protein-coupled receptors (GPR41 and GPR43, also known as free fatty acid receptors FFAR3 and FFAR2, respectively) on enteroendocrine L cells. This binding results in the activation of heterotrimeric G proteins.^99^ One of the G proteins, Gi/o, stimulates the enzyme adenylyl cyclase, which increases the cAMP levels. This is followed by the binding of cAMP to the PKA enzyme and its activation, which then phosphorylates transcription factors and target proteins, subsequently upregulating the proglucagon gene, which then produces active glucagon. This cascade of metabolic events involving intracellular signaling eventually results in the secretion of GLP-1 from the L cells into the bloodstream.^100^ The circulating GLP-1 binds to the GLP-1 receptor in the β cells and activates the Gs protein pathway, resulting in subsequent stimulation of adenylyl cyclase, cAMP, and the cAMP-regulated guanine nucleotide exchange factor, Epac,^100^ which sensitizes multi-subunit calcium channels on the endoplasmic reticulum, causing conformational changes resulting in the opening of the calcium channels and calcium releases in the cytosol.^101^ Besides this, increased ATP released due to glucose metabolism closes KATP channels and opening of L-type voltage-dependent calcium channel, which causes an influx of calcium in the cytosol. This calcium causes exocytosis of insulin-containing vesicles, which release insulin upon diffusion.^100^ These pathways explain the role of SCFAs in improving glucose metabolism. In alignment with these findings, Wu et al^102^ investigated the relationship between GDM and potential microbial biomarkers in the Chinese pregnant population. The study revealed that SCFA pathways are impaired in GDM pregnancies, which, in turn, affects the gut microbial composition.^102^ The species-level β diversity differed significantly between control participants and those with GDM, with *L. casei* being a discriminant microbiome in non-GDM pregnancies.^102^ Most human trials investigating the role of probiotics and prebiotics in the management of GDM did not quantify the SCFA production, despite its potential role as a key mechanistic pathway. In contrast, an animal model study^103^ provided experimental evidence by demonstrating the antidiabetic impact of a 6-week probiotic intervention comprising 10 *Lactobacillus* strains (1 × 10^10^ CFU mL^–1^) and 4 Saccharomycetes strains (1 × 10^8^ CFU mL^–1^), all isolated from fermented camel milk. The intervention enhanced SCFA production, likely due to an increase in SCFA-producing bacteria, thereby improving intestinal barrier integrity, upregulating GLP-1 production, and enhancing glucose metabolism in mice with T2DM.^103^ The metabolic benefits are particularly significant in the context of GDM, which is not only characterized by insulin resistance and disrupted glucose regulation but also by persistent low-grade inflammation.

In a hyperglycemic state, subsequent glycolysis causes higher pyruvate production, which diffuses into mitochondria and enters the Krebs cycle. This causes enhanced input of reducing equivalents like nicotinamide adenine dinucleotide and reduced flavin adenine dinucleotide into mitochondria, resulting in overproduction of reactive oxygen species,^104^ activating inflammatory cascades that result in pro-inflammatory gene expression. In recent years, probiotics have gained popularity due to their anti-inflammatory effects, with several emerging reviews attempting to highlight the anti-inflammatory potential of probiotics in different conditions; however, there are limited RCTs exploring the use of probiotics in alleviating inflammation in GDM. Results from our scoping review revealed that probiotic intervention substantially reduces serum hs-CRP, IL-6, and TNF-α with significant improvements (large effect size) compared with a placebo. Similar conclusions have been drawn from the combined analysis of 7 studies involving 665 participants, the results of which strongly supporting the role of probiotics in improving inflammatory biomarkers in GDM.^105^ The analysis, however, included studies published until 2020. Consistent findings were reported in patients with prediabetes in a pilot trial in which the 12-week-long intake of *Lactiplantibacillus plantarum* suppressed TNF-α, IL-1β, and IL-6.^106^ The use of probiotics has long been known to modulate gut microbes and intestinal physiology via different mechanisms.^107^ They enhance the expression of proteins, including ZOs and transmembrane protein occludin, both of which provide strong architectural integrity to tight junctions; dysregulation thereof indicates a disturbed homeostasis in the gut.^108^^,^^109^ Gut microbiome has an affinity to change with diet while simultaneously mediating the effects of diet on gut health^110^ through complex interactions. Animal model studies have also demonstrated that probiotics containing *Lactobacillus* species reduce the abundance of LPS-producing bacteria such as Pseudomonadota (formerly Proteobacteria) and increase SCFA-producing bacteria, including *Roseburia*, which enhance gut integrity and prevent translocation of LPS into circulation, thereby reducing systemic inflammation.^111^ Such microbial alterations may play a role in the pathophysiology of diabetes, highlighting the potential of targeting gut microbiota in therapeutics. Notably, supplementation of the probiotic-fermented milk containing the *L. casei* strain Shirota for a period of 2-5 weeks has been shown to positively influence the gut microbiota, including correcting dysbiosis, improving intestinal environment, and enhancing microbial immunity in individuals without diabetics ^112–114^ A minimum of 4 weeks of probiotics supplementation with *Lactobacillus* species can lead to significant alterations in gut microbiota, which, in turn, may help prevent insulin resistance.^115^

Proinflammatory cytokines such as TNF-α impair insulin signaling by disrupting the insulin receptor substrate phosphorylation. Species like *Lactiplantibacillus plantarum* reduce TNF-α, IL-6, and IL-8 levels while increasing anti-inflammatory cytokines like IL-4, mitigating chronic low-grade inflammation.^111^ Impaired glucose metabolism in T2DM is associated with specific shifts in the gut microbial community, particularly an increase in Betaproteobacteria and decrease in Bacillota (formerly Firmicutes), particularly Clostridia.^116^ Su et al^117^ reported an increased abundance of *Bacteroides,* and *Citrobacter desulfovibrio* in women with GDM relative to normoglycemic women during the second trimester of pregnancy. Additionally, an increased proportion of *Prevotella* was found during the postpartum period in women who had GDM compared with women with no GDM.^118^

The consistent positive impact of probiotic supplementation across diverse populations, including individuals with GDM and with T2DM, highlights its potential as a valuable therapeutic approach. Despite the physiological differences between GDM and T2DM, the ability of probiotics to improve glucose metabolism, modulate inflammation, and hence, gut microbiota underscores their broad applicability. This consistency across varying conditions strengthens the evidence for probiotics as an effective intervention in managing glucose-related metabolic challenges. Moreover, to make our results more stringent, we discussed only trials that excluded women with preexisting diabetes. All participants developed glucose intolerance during pregnancy, reducing heterogeneity in findings and ensuring that the metabolic effects of probiotic intervention are assessed specifically in the context of GDM. However, we still observed discrepancies among the different studies despite using matching species that can be attributed to different sample sizes, duration of intervention, as well as dosage administered. In addition to these variables, patient compliance with the intervention is 1 major factor that could have resulted in contrasting findings, because reported patient compliance in clinical trials ranges from 43% to 78%.^119^ In the trials included in this scoping review, adherence differed in each study and was not uniform.

Dietary patterns were not standardized across the trials, and participants maintained their habitual diets. Most studies did not report baseline dietary intake, limiting assessment of factors like fiber or probiotic content that affect metabolic outcomes. Diet can modulate the gut microbiome over time^120^, influencing probiotic efficacy. Baseline microbial composition, a key factor in response to probiotics, was not considered in the included studies.^121^ Emerging evidence shows that existing gut microbiota can affect probiotic effectiveness,^122^ with individuals having lower baseline diversity potentially benefiting more. Conversely, those with already rich microbiota may have reduced metabolic response to probiotics. Taken together, these findings highlight both the promise and the complexities involved in probiotic intervention for GDM. The findings and critical evaluation in this review will aid in preparing the ground for future research, specifically for the pregnant population. In comparison to previous reviews conducted to summarize the evidence on the efficacy of probiotic supplementation to alleviate GDM, this scoping review has followed a rigorous search strategy with a statistical investigation focusing on effect sizes for each parameter measured concerning glucose metabolism and inflammation, while discussing a broader scope.

### Future Research

Despite all the benefits to the host, several gaps exist that can be addressed in future research. A personalized approach to preventive medicine with respect to these probiotics is lacking in the prevention and treatment of GDM. Studies using a multiomics approach to understand how probiotics affect gene expression and signaling pathways, as well as the identification of metabolites and biomarkers related to glucose metabolism and inflammation, will help predict the susceptibility of women to GDM. A growing body of probiotics research has used encapsulated forms, and clinical trials on probiotic-rich foods remain relatively scarce compared with those with capsules, likely due to ease of administration or intake. Efficacy comparison between encapsulated probiotics and fermented foods will become more feasible as more data emerge from the trials on fermented food as an intervention. Beyond the use of probiotics, recent evidence highlights the beneficial role of diet-based interventions in the management of GDM. A clinical trial was designed to demonstrate the impact of increased fiber and fermented foods on maternal gut microbial diversity during pregnancy and the influence on the early development of the infant microbiome.^123^ Similarly, supplementation with whole blueberries and soluble fiber in pregnant women with obesity significantly improved glycemic control, reduced inflammation, and prevented excess gestational weight gain.^124^ These studies highlight a growing recognition of the gut-metabolic axis in gestational health and suggest the potential of nonpharmacological strategies such as prebiotics and fermented foods in managing GDM.

Given that the prevalence of GDM varies with region, evidence from diverse populations accounting for variation in different lifestyles and environmental factors, is crucial to understanding the application of such an intervention from a global perspective. Furthermore, evidence is limited in relation to strain-specific microbes that are beneficial yet resilient in the gut and can survive the enzymes and fluids in the gut. Because a multitude of RCTs have chosen multistrain probiotics as an intervention, it is difficult to differentiate exactly which probiotic strain exerts what effect on the outcome of interest. Hence, probing individual strains is important. In addition, research should be directed toward identifying a standardized probiotic formulation. Last, limited evidence exists regarding the impact of probiotic supplementation on inflammatory biomarkers during pregnancy, which warrants further research focusing on their role in modulating inflammation.

## CONCLUSION

Our review highlights the positive role of *Lactobacillus* and *Bifidobacterium* species in improving biomarkers of glucose metabolism and inflammation and in enhancing overall gut health in women with GDM. Though the studies differed in probiotic doses and strains used, the overall outcomes consistently indicated a beneficial effect. The review also shows that both probiotic capsules and fermented foods containing probiotic strains are effective in mitigating inflammation and balancing glucose levels and, hence, can be used as a prophylactic and therapeutic measure for women with GDM or in normoglycemia during pregnancy to prevent the incidence of GDM. Despite a huge volume of evidence on the potential of probiotics in alleviating hyperglycemia and inflammation during pregnancy, there remains variation among studies with respect to a standardized approach. Although the existing body of evidence supports the beneficial effects of probiotics supplementation in GDM, it is important to recognize the geographical bias in the evidence due to the majority of studies being conducted in 1 country, which limits the generalizability of findings. To build a more comprehensive understanding of probiotics’ potential in GDM, RCTs should be conducted across diverse populations and regions to validate the current evidence.

## Supplementary Material

nuaf238_Supplementary_Data
